# Alveolar Epithelial Type II Cells Activate Alveolar Macrophages and Mitigate *P. Aeruginosa* Infection

**DOI:** 10.1371/journal.pone.0004891

**Published:** 2009-03-23

**Authors:** Shibichakravarthy Kannan, Huang Huang, Drew Seeger, Aaron Audet, Yaoyu Chen, Canhua Huang, Hongwei Gao, Shaoguang Li, Min Wu

**Affiliations:** 1 Department of Biochemistry and Molecular Biology, University of North Dakota, Grand Forks, North Dakota, United States of America; 2 Division of Hematology/Oncology, Department of Medicine, University of Massachusetts Medical School, Worcester, Massachusetts, United States of America; 3 State Key Laboratory for Biotherapy, West China Hospital, Sichuan University, Chengdu, China; Oregon Health & Science University, United States of America

## Abstract

Although alveolar epithelial type II cells (AECII) perform substantial roles in the maintenance of alveolar integrity, the extent of their contributions to immune defense is poorly understood. Here, we demonstrate that AECII activates alveolar macrophages (AM) functions, such as phagocytosis using a conditioned medium from AECII infected by *P. aeruginosa*. AECII-derived chemokine MCP-1, a monocyte chemoattractant protein, was identified as a main factor in enhancing AM function. We proposed that the enhanced immune potency of AECII may play a critical role in alleviation of bacterial propagation and pneumonia. The ability of phagocytosis and superoxide release by AM was reduced by MCP-1 neutralizing antibodies. Furthermore, MCP-1^−/−^ mice showed an increased bacterial burden under PAO1 and PAK infection vs. wt littermates. AM from MCP-1^−/−^ mice also demonstrated less superoxide and impaired phagocytosis over the controls. In addition, AECII conditioned medium increased the host defense of airway in MCP-1^−/−^ mice through the activation of AM function. Mechanistically, we found that Lyn mediated NFκB activation led to increased gene expression and secretion of MCP-1. Consequently Lyn^−/−^ mice had reduced MCP-1 secretion and resulted in a decrease in superoxide and phagocytosis by AM. Collectively, our data indicate that AECII may serve as an immune booster for fighting bacterial infections, particularly in severe immunocompromised conditions.

## Introduction

Alveolar type II epithelial cells (AECII) maintain alveolar integrity by forming the alveolar barrier, producing surfactants and repairing injured type I epithelium [1]. Recently, several reports indicate that AECII may be an integral part of the lung innate immunity, acting to intensify the function of dendritic cells [2] and alveolar macrophages [3]. AECII may also perform other important functions such as cytokine secretion [4], antigen presentation [5] and enhancement of macrophage phagocytosis through surfactant proteins such as SP-D [6]. However, the role of AECII in infection immunity and underlying mechanism is largely unknown. Understanding the additional immunological role of AECII is important because professional immune cells such as alveolar macrophages (AM) in some cases rarely succeed in eradicating pathogens on their own. This is the case with *Pseudomonas aeruginosa*, an opportunistic gram-negative bacterium accounting for 10.1% of nosocomial infections. Clinical reports have shown that *P. aeruginosa* infects about 97.5% of Cystic Fibrosis children at age of 3 years [7]. *P. aeruginosa* infection is a main clinical problem in various immunodeficiency conditions such as HIV, severe burns, and cancers. This bacterium exhibits resistance to conventional antibiotics through a variety of virulence factors and there are no effective vaccines, making it difficult to eradicate once it colonizes the respiratory tract [8], [9].

Previous studies demonstrated that AECII cells can stimulate AM immunity in the lung of either mice or rats to boost the host defense against *P. aeruginosa* [4]. The caveats are that the studies have focused on whole animals to identify the cell types responsible for secreting chemokines by co-localization techniques. Also, the specific cell type involved in the immune activity has not been clearly defined. Although the main chemotactic factor released from AECII is likely to be MCP-1 [4], it is hard to prove *in vivo* whether AECII are the actual secreting cells. Also, the specific role of MCP-1 derived from AECII is less clear. A recent study suggested that isolated human AECII may secrete chemokines such as MCP-1 at higher amounts than AM primed with lipopolysaccharide (LPS) [10]. However, the specific immune response by characteristically defined AECII to *P. aeruginosa* infection remains to be determined. Furthermore, the underlying mechanism governing AECII mediated host immunity and regulatory factors involved in secretion of MCP-1 are not clearly established. Thus, it is necessary to investigate and fully establish the immune role of AECII.

To determine the immunological function of AECII, we chose to investigate if AECII have a role in activating AM in response to *P. aeruginosa* infection. We used our primary cell culture models as well as mouse models including knockout mice to identify the signaling proteins that may regulate AM function and by identifying the cytokine products derived from AECII. We have shown here the importance of MCP-1 secreted by AECII in the activation of AM. Using MCP-1^−/−^ mice, we also confirmed that MCP-1 has a crucial activity in increasing AM functionality through enhancing phagocytosis, increasing superoxide production, balancing inflammatory response and thereby optimizing host defense. In addition, we delineated the mechanism that regulates MCP-1 secretion in AECII, in which we found that Lyn, a critical Src family member, can increase MCP-1 secretion by activating the NF-κB pathway.

## Results

**Figure 1:**
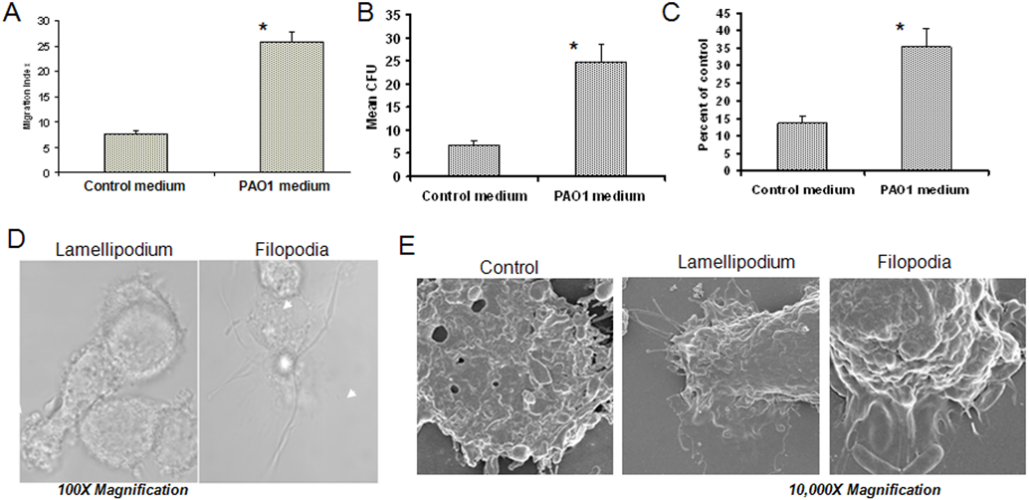
Conditioned medium from AECII by PAO1 infection activated AM (12 h). (A) Increase of AM migration as determined by chemotaxic chamber methods (see Material and Methods). Migration index was calculated based on conditioned medium or control medium. (B) Increase in phagocytosis of AM as detected by CFU (colony forming units). (C) Increase in superoxide production of AM analyzed by H2DCF fluorescence (Molecular Probes). (D) The morphology of the activated AM is shown typically with lamllipodium and filopodium by confocal microscopy. (E) The morphology of the activated AM is further evidenced by high resolution and magnification Scanning Electron Microscopy (SEM). Statistical analysis was done by comparing mean individual values versus controls using student's *t* test, *p<0.05 (95% Confidence Interval, CI). The results are representative of three experiments.

Our objective was to test whether AECII in a culture system can boost the immune function of AM. Through co-culturing macrophages with lung epithelial cells, macrophages were significantly activated, demonstrating multiple functional activities including enhanced migration and actin reorganization (Figure S1A, B). To investigate whether secreted (soluble) substances are major mediators, we collected a conditioned medium from primary mouse AECII following PAO1 infection for 1 h and added to primary AM culture. After 24 h, AM activity was detected by three measurements: migration (Figure 1A), phagocytosis (Figure 1B) and superoxide production (Figure 1C). Our data indicates that the three AM function parameters were significantly increased by the AECII conditioned medium compared to the control medium from AECII without infection (*P<0.05*, Student's *t* test). The activated AM also showed more significant morphological changes with membrane projections such as lamellipodia and filopodia by confocal microscopy (Figure 1D). Moreover, we demonstrated apparent morphology alterations by scanning electron microscopy (SEM) (Figure 1E). Although we found that AECII have a role in activating AM, it is possible that AM themselves can be activated by *P. aeruginosa*, which enhances the function of resting AM through an autocrine loop. Thus, we evaluated whether the activated AM can bolster immunity by stimulating other naïve AM. We co-incubated resting AM with a conditioned medium that was derived from AM following *P. aeruginosa* time course infection. As expected, we have found that the AECII conditioned medium indeed showed greater increases in AM migration and superoxide production compared to the AM-conditioned medium, particularly at earlier times (6 h, Figure S1C). However, AM also secreted comparable MCP-1 possibly through autocrine activation at 24 h.

**Figure 2:**
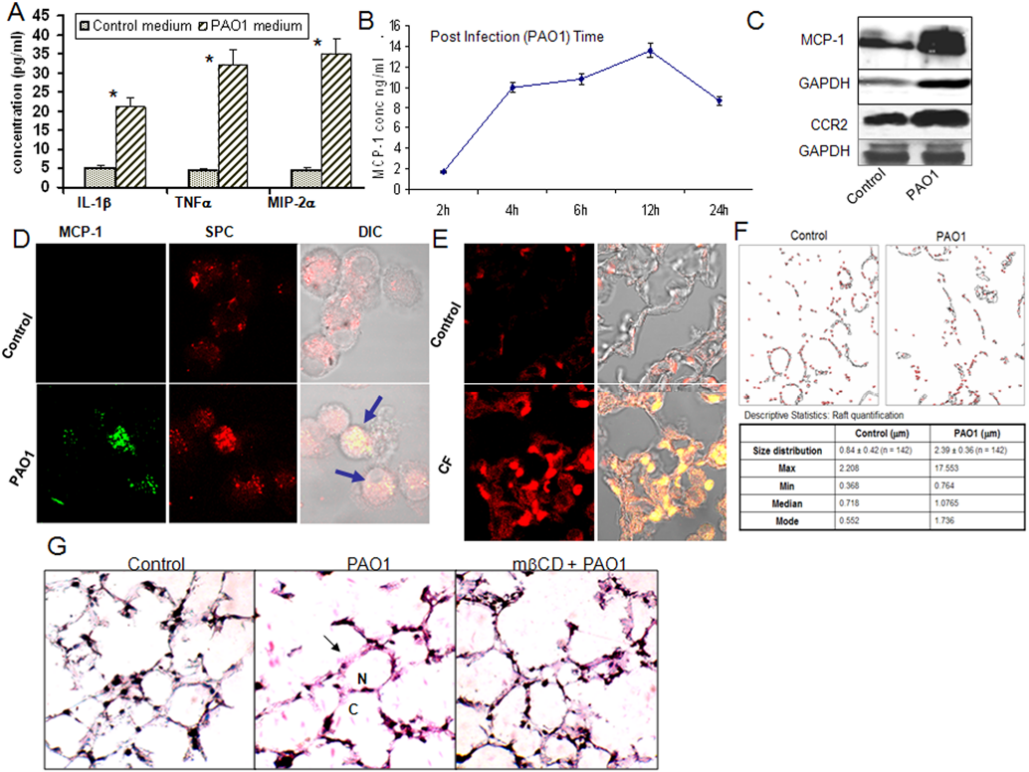
MCP-1 from AECII was a major regulator by PAO1 infection. (A) Increase of various proinflammatory factors at 24 h following stimulation with the conditioned medium from AECII infected by PAO1. (B) MCP-1 was significantly increased in a time dependent manner by the conditioned medium. (C) Both MCP-1 and its receptor of CCR2 were increased in AECII by PAO1 infection detected by western blotting. (D) Primary AECII secreted MCP-1 that was colocalized (arrows) with SP-C (a specific marker of AECII) as analyzed by immuno-fluorescence (anti-pro-SP-C from Dr. Jeffrey Whitsett). (E) Increase of MCP-1 expression in the AECII cells in human samples from Cystic Fibrosis (CF) patients (one of the 6 representative) stained by immunofluorescence. (F) Lipid raft quantification using Image J software. The colocalized pixels were identified and threshold defined vs. background. The image was converted into binary format and the “analyze particle tool” used to identify raft ranging in size of 15–255 pixels (correlates with 50–200 nm). (G) Blockade of lipid rafts with mβCD significantly reduced the secretion of MCP-1 in the lung vs. the control. The frozen sections for the lung tissue of C57BL6 mice (5 mice/group) were stained by anti-MCP-1 antibodies from Santa Cruz (arrows showing the positive staining). The results are representative of three experiments.

To investigate how AECII can activate AM, we studied the possible involvement of various cytokines and chemokines. Thus we performed an *in vitro* experiment with isolated primary mouse AECII to measure cytokines in the conditioned medium of AECII infected by *P. aeruginosa*, which showed a significant increase in MCP-1, IL-1β, MIP-2α, and TNF-α (Figure 2A, B). We also showed that MCP-1 can transmit cellular signals to AM (4), and may be a dominant cytokine in cultured AECII cells. The secretion of MCP-1 is time dependent (Figure 2B). Also, we detected increased expression of MCP-1 and CCR2 (MCP-1 receptor) expression on AECII cells by western blotting (Figure 2C). We further confirmed that the MCP-1-secreting cells expressed SP-C (a marker of AECII) by co-localization with confocal microscopy (Figure 2D). Similar results showing cytokine secretion by AECII were also found in murine lung type II cell line MLE-12 cells [11] (Figure S2A). We then infected mice to investigate MCP-1 secretion in response to *P. aeruginosa* infection in *“in vivo”* conditions [12]. C57BL6 mice (8 weeks female, 5 mice per group) were infected with PAO1 intranasally, and broncho-alveolar lavage (BAL) was performed 18 h post infection. MCP-1 level was found to be increased as measured by immunohistochemistry in lung tissue, and PAO1 infected mice showed a significant increase in MCP-1 compared to uninfected controls (Figure 2G). Furthermore, the MCP-1 expression was demonstrated on AECII cells of human lung tissues from CF patients with *P. aeruginosa* infection (Figure 2E), suggesting that MCP-1 actually participated in host defense in infected humans. We showed that lipid rafts in AECII are reorganized into aggregates (platforms for cell signaling) following acute infection by *P. aeruginosa* [12], which is consistent with several studies regarding *P. aeruginosa* or other microorganisms [13]–[15]. *P. aeruginosa* has type three secretion system (T3SS) exoenzymes ExoS, ExoT, ExoU and ExoY, among which ExoS and ExoT are similar in the sequence and function for ADP ribosyltransferase and GTPase-activation protein activities [16]. The raft aggregates are differentially induced by various T3SS mutants or a pili deficient mutant [12]. Since there were some rafts outside the *P. aeruginosa*, we further quantified raft aggregates using Image J software and showed that *P. aeruginosa* infection induced strong raft aggregates, which were also associated with *P. aeruginosa* (Figure 2F). In addition, controls without infection did not show raft aggregates, thus the aggregates of lipid rafts may be a specific response to *P. aeruginosa*. Next, we used various inhibitors to define the raft pathway in *P. aeruginosa* adhesion and internalization into AECII. Among the various inhibitors we have tested, we found that the lipid raft inhibitor (mβCD), Lyn tyrosine kinase inhibitor (PP2), Akt inhibitor, and NF-κB inhibitor effectively blocked adhesion and internalization of *P. aeruginosa*, whereas the ceramide inhibitor (blocking sphingolipid pathway) only reduced internalization without affecting adhesion (Figure 2G, Table 1 and Figure S2B). This data suggests that ceramide and cholesterol may differentially impact *P. aeruginosa* adhesion and internalization. To further dissect the mechanism, we intranasally instilled cholesterol chelator mβCD into mouse lungs and found a reduction in bacterial internalization and MCP-1 secretion was also partially hindered (Figure 2G).

**Table 1:** Inhibition of adhesion and internalization of bacterium.

Regent	Adhesion	Internalization
Control	26	0.89
mβCD	2.2	0.07
Ceramide inhibitor	18	0.13
PP2	4.2	0.28

* Raft blockers (mβCD cholesterol chelator, and ceramide inhibitor [Racemic] 3 mM Alexis) hampered PA internalization. PP2 is Lyn inhibitor.

**Figure 3:**
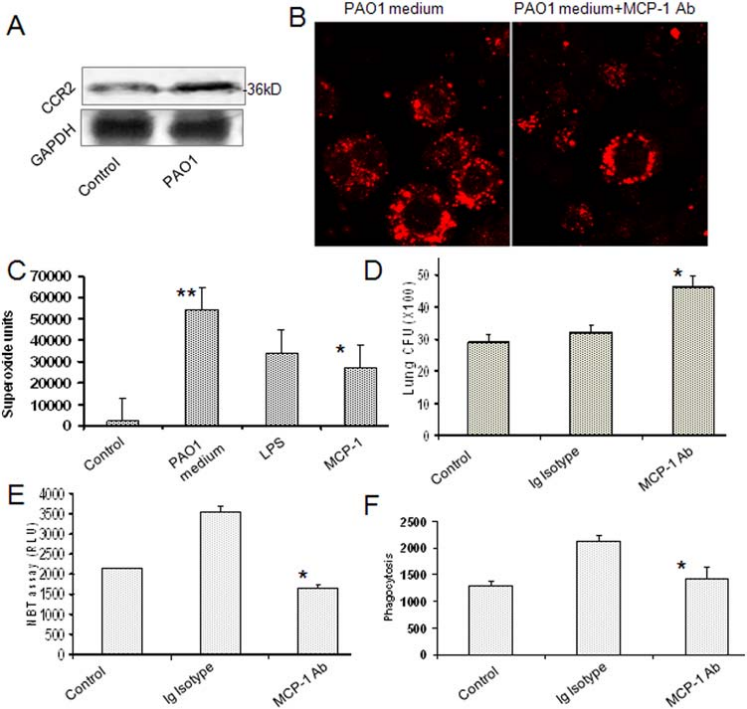
MCP-1 receptor was increased along the enhanced immune function and MCP-1 antibody alleviated the infection. (A) The receptor of MCP-1 (CCR2/CKR2) was also increased in AECII by PAO1 infection detected with western blotting. (B) CCR2 expression following PAO1 infection was reduced by pre-incubating with MCP-1 antibodies (30 min) and determined by immunofluorescence. (C) Increase in superoxide release was measured using H2DCF by direct addition of the commercially available MCP-1 peptide. (D) BAL CFU from mice was increased by neutralizing antibodies against MCP-1 before PAO1 infection. (E) Superoxide was reduced in the AM from mice following instillation of MCP-1 antibodies as measured by NBT assay. (F) The AM from the same mice also demonstrated decreased phagocytosis ability. Statistical analysis was performed as above (** *p*<0.01, 99% CI). The results are representative of three experiments.

The expression of CCR2 (MCP-1 receptor) was increased in AM treated with AECII conditioned medium as determined by western blots (Figure 3A), indicating a functional involvement of this receptor in the pathway. Next, blocking of MCP-1 with neutralizing antibodies for 30 min before infection also down-regulated CCR2 compared to no antibody controls using fluorescent microscopy (Figure 3B). To further confirm the role of MCP-1, we added commercially available purified MCP-1 peptide (Calbiochem) directly to AM and found direct correlation between MCP-1 presence and AM activation, including superoxide production (Figure 3C). Furthermore, by blocking with MCP-1 neutralizing antibodies for 30 min before PAO1 infection, we observed that the mice showed more severe infection with increased bacterial burden (CFU/1 mg lung homogenates) compared to that in the wt mice (Figure 3D). In addition, the MCP-1 antibodies reduced superoxide production (Figure 3E) and phagocytosis by AM (Figure 3F) against the Ig isotype control. To ascertain the immune activity of AECII, we determined the immune symbolic markers, such as IL-12R and MHC Class II antigen by immunofluorescence. We showed that activated AECII expressed enhanced levels of MHC class II antigen as well as IL-12R and IL-17R (Figure S3A, B).

**Figure 4:**
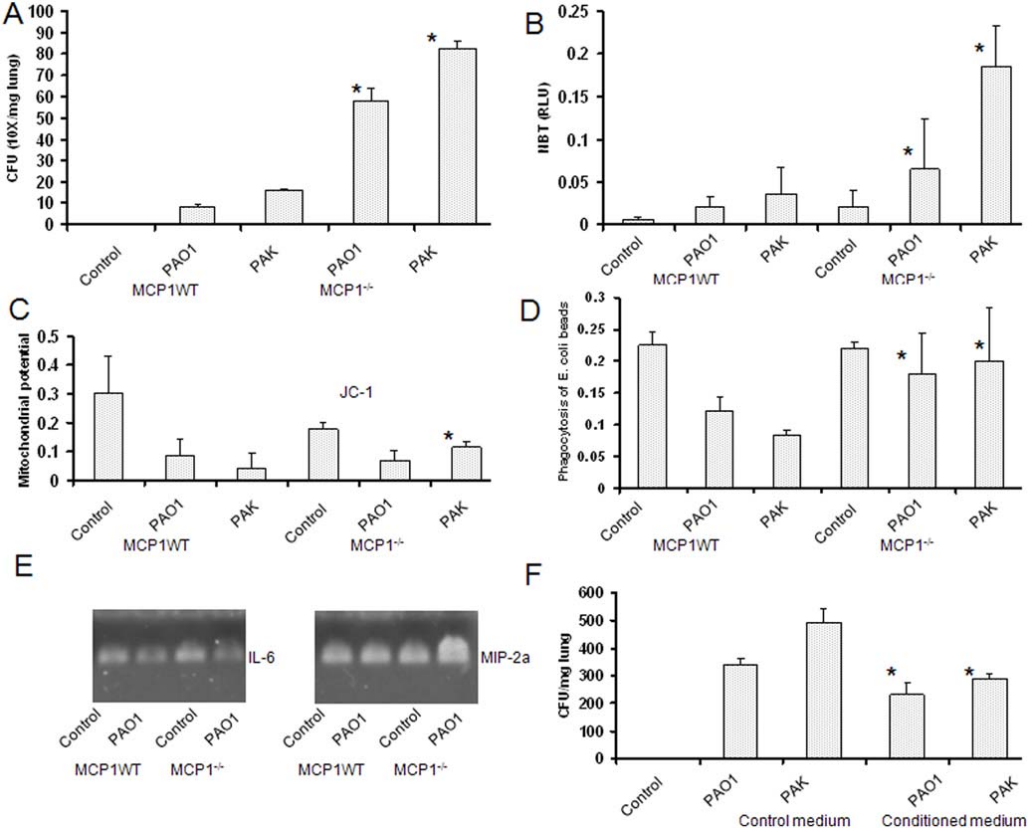
MCP-1^−/−^ mice demonstrated functional involvement with P. aeruginosa infection. (A) MCP-1^−/−^ mice showed more severe infection by *P. aeruginosa* (strains PAO1 and PAK) with an increase in bacterial burdens. Data were derived from a group of 5 infected and control mice (same in the figure). (B) Superoxide production was significantly altered in AM from MCP-1^−/−^ mice following infection identified by NBT assay. (C) A significant increase in apoptotic cell death was observed in MCP-1^−/−^ mice by bacterial infection. The data identified the loss of mitochondrial potential in MCP knockout mice, indicating increased cell apoptosis. (D) An increase opsonic phagocytosis with *E. coli* particles was found in AM derived from MCP-1^−/−^ mice following infection. (E) An increase in inflammatory chemokine MIP-2α in the MCP-1^−/−^ mice after PAO1 infection. The mRNA expression is shown in an agarose gel using semi-quantitative RT-PCR. (F) Conditioned medium partially restored the immune defense against *P. aeruginosa* infection in MCP-1^−/−^ mice. Statistical analysis was as above. The results are representative of two experiments.

To further determine the role of MCP-1 as an immune stimulator, MCP-1 deficient mice (MCP^−/−^ mice) were used to study MCP-1 effects on AM immune function and physiological role during *P. aeruginosa* infection. This can also distinguish the relative contributions of MCP-1 to immune defense against *P. aeruginosa* infection. We found that MCP-1^−/−^ mice showed increased bacterial burden in the lung (Figure 4A). A significant decrease in the superoxide production in AM was also noted (Figure 4B). Furthermore, the lung from MCP-1^−/−^ mice showed increased lung cell death (showing decreased mitochondrial potential) following infection by *P. aeruginosa* (Figure 4C). Lung injury was also present as wet/dry ratio was increased by infection in the mice (data not shown). We also showed that isolated AM from MCP-1^−/−^ mice after bacterial infection had increased ability to phagocytose opsonized-*E coli* particles (Figure 4D), indicating that the AM have not been saturated in uptaking bacteria (probably due to reduced migration) during *in vivo* infection. Interestingly, we noted that the lung injury is correlated with the increase in a number of pro-inflammatory factors such as MIP-2α (but probably also due to the loss of MCP-1) than the wild-type control (Figure 4E). Also, there was an increased neutrophil infiltration in the BAL (not shown). These data suggest that MCP-1 may be crucial in maintaining a fine balance between pro-inflammatory and anti-inflammatory cytokines, which is important for combating bacterial invasion as well as minimizing acute lung injury. Also, we instilled the conditioned medium into MCP-1^−/−^ mouse lungs, which increased the host defense compared to the control medium (Figure 4F). These data critically confirm that MCP-1 is a potent immune regulator and inflammation controller in the airway spaces.

**Figure 5:**
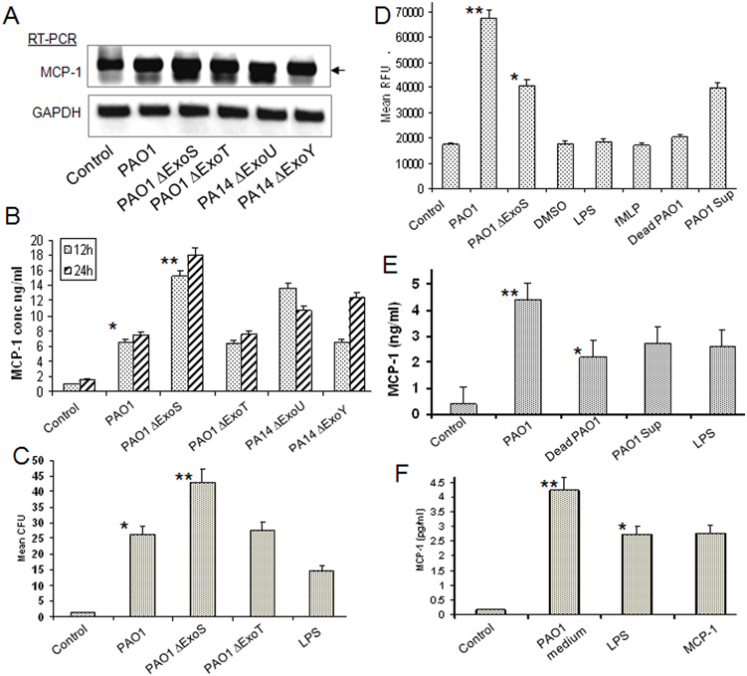
Exoenzymes were involved in MCP-1 secretion. (A) ΔExoS strain induced increased MCP-1 mRNA and protein expression by MLE-12 lung epithelial cells assessed by RT-PCR and ELISA (B). (C) ΔExoS strain resulted in increased phagocytosis by AM than PAO1 wt. (D) ΔExoS strain induced decreased superoxide in AM (PAO1 sup: PAO1 supernatant; fMLP 5 µM from Sigma). (E) Less potency by dead PAO1, PAO1 supernatant and LPS (*P. aeruginosa* lipopolysaccharide, serotype 10, 200 ng/ml, Sigma) in inducing MCP-1 than live PAO1. (F) MCP-1 alone also induced MCP-1 secretion in primary AECII. Statistical analysis was performed as above. The results are representative of two experiments.

Because raft signaling is partially dependent on T3SS, we attempted to define whether deletion in a single exoenzyme impacts the MCP-1 secretion by AECII. PAO1 wt and several toxin deficient strains (PAO1 ΔExoS, ΔExoT, PA14 ΔExoU and ΔExoY) were employed to examine the effect on AECII cells. Our data indicates that the various T3SS mutant strains influenced secretion of MCP-1 compared to PAO1 wt (except ΔExoT), the highest inducer being ΔExoS as determined by RT-PCR and ELISA (Figure 5A, B). Consequently, the conditioned medium from mutant strain ΔExoS induced greater AM activation (phagocytosis) than PAO1 wt (Figure 5C). By contrast, ΔExoS strain resulted in lesser superoxide in AM than PAO1 wt strain (Figure 5D). One possible explanation for this result is that loss of a particular exoenzyme may reduce the bacterial invasive ability, permitting the host to mount a better defense. The controls with normal medium, DMSO (for inhibitor dilution) and PAO1 supernatant induce measurable but lesser response than that of live PAO1 (Figure 5D). We next examined whether live *P. aeruginosa* is necessary to induce MCP-1 secretion by AECII and to turn the cells immunologically more potent. Using dead PAO1 (heating at 60°C for 1 h), supernatant, LPS and live PAO1 wt (in the same amount of bacteria and same number of cells), we compared differences in MCP-1 secretion during infection of AECII. We found that live PAO1 increased MCP-1 secretion more so than did dead PAO1, supernatants, and LPS. The conditioned medium from live infection also induced the strongest activation of AM (Figure 5E). Thus, it is likely that live bacterial infection initiated stronger stimulation of AECII secretion than dead bacterium or its components.

Since Src tyrosine kinase Lyn is located in the inner cytoplasm membrane of the cell, in the general proximity to lipid rafts, we investigated the role of several members of this family and discovered that Lyn (p53/56) played a major role in *P. aeruginosa* infection. We have demonstrated that Lyn was activated in A549 cells following exposure to *P. aeruginosa* [12]. Lyn phosphorylation was measured by immunoblotting with phosphor-Src antibodies on immunoprecipitated Lyn protein from cell lysates. Pre-treatment of AECII with 5 nM PP2 (synthetic inhibitor for Lyn) blocked MCP-1 secretion (Figure S2B).

**Figure 6:**
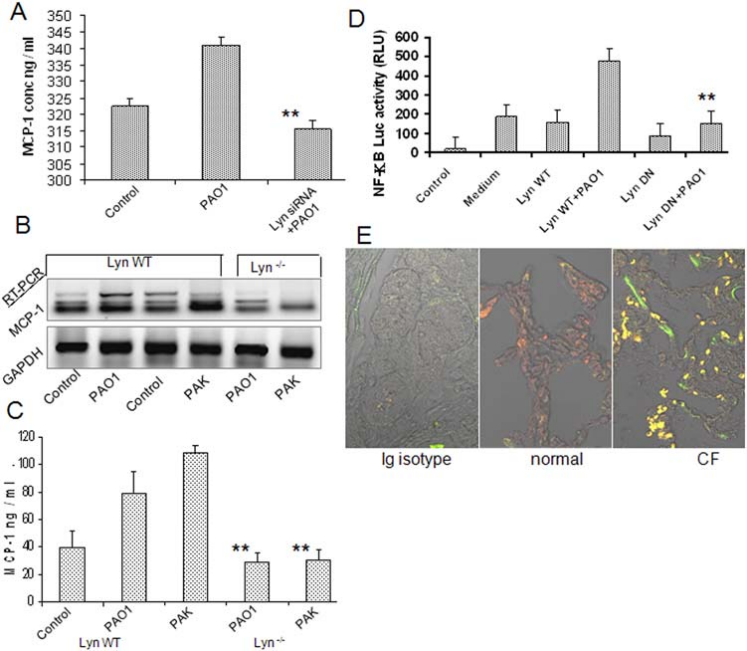
Lyn regulated MCP-1 production by activating NF-κB. (A) MCP-1 secretion was abolished by Lyn siRNA transfection in AECII cells. (B) Decrease in MCP-1 expression in the lungs. (C) Decrease in MCP-1 expression secretion in BAL fluid (bronchoalveolar lavage) in Lyn^−/−^ mice by *P. aeruginosa* infection. Data were representative of two experiments with a group of 5 mice (infected and control). (D) Dominant negative Lyn construct transfection (DN = LynK275D) reduced NF-κB translocation in AECII as determined by a Luciferase NF-κB reporter assay. (E) Increased expression and activation of Lyn in human lung infected with *P. aeruginosa*. Tissues were from CF patients and normal controls (showing representative of 6 samples). Statistical analysis was done as above. The *in vitro* results are representative of three experiments.

To elaborate the role of Lyn in MCP-1 production, Lyn siRNA transfection was performed to assess the effect of Lyn on *P. aeruginosa* invasion (transfection efficiency >95%) [17], and our data show that MCP-1 was markedly decreased by Lyn siRNA (Figure 6A). Importantly, the role of Lyn was confirmed using Lyn^−/−^ mice, demonstrating significantly reduced MCP-1 by PAO1 and PAK infection vs. Lyn wt mice as determined by mRNA expression (Figure 6B). Reduction of MCP-1 secretion was also observed in Lyn^−/−^ mice as determined by ELISA (Figure 6C), whiles there is no difference between wt and KO mice in non-treated condition (data not shown). To probe downstream effectors in this pathway, we investigated NF-κB activation in response to *P. aeruginosa* infection. We evaluated this signaling process and our data showed that blocking of Lyn using dominant negative transfection of LynK275D construct [12] significantly reduced the NF-κB nuclear translocation compared to Lyn wt control transfectants (not shown). Using NF-κB luciferase plasmid, we found a pronounced increase in luciferase activity with conditioned medium obtained from PAO1 infected AECII cells, but this was blocked by LynK275D transfection (Figure 6D). We also observed similar Lyn-K275D-based inhibition in functional roles of AM. These findings suggest that Lyn signaling is linked to both the secretion of AECII and its functional role of AM. Finally, we also demonstrated that Lyn overexpression and increased phosphorylation in the human lung tissue of CF patients against the normal control (Figure 6E). Our data indicate that Lyn activation might have occurred during CF disease process, thus it is reasonable to propose that Lyn may serve as a therapeutic target for controlling the disease.

**Figure 7:**
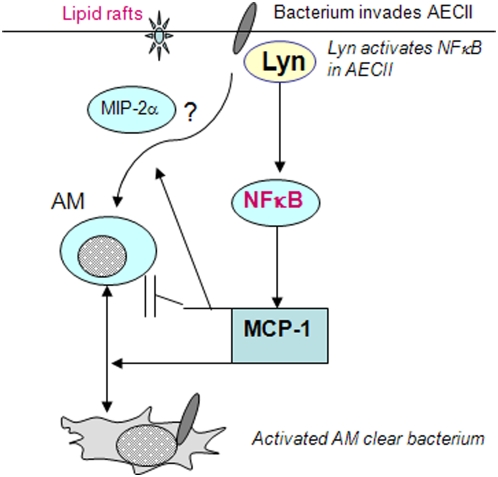
Diagram delineates a pathway involved in the AECII immune role against P. aeruginosa infection. AECII cells initiate an immunity to boost AM anti-infection function. The chemokine MCP-1 seems a crucial factor in this cell-cell cross-talk, which may be initiated by lipid rafts and regulated by Lyn. While boosting MCP-1 production through NFκB pathway, Lyn may contain excessive production of MIP-2α as another mechanism to keep the inflammation response in control.

The AECII – AM cross-talk is presented in a simplified model (Figure 7). Bacterial infection activates AECII cells that secrete chemokines (MCP-1). MCP-1 transmits signals to activate AM, *which* migrate towards infection sites and eliminate the pathogen. Lyn and lipid rafts may regulate MCP-1 and AM activation. In addition, the host defense may be balanced through the fine regulation by both Lyn and MCP-1.

## Discussion

Previous reports, either from animal studies or from work with lung epithelial cells, indicate that epithelial cells can secrete cytokines to increase AM immune function in combating *P. aeruginosa* infection [2], [4]. Our studies use both isolated AECII mouse cells and animals (including knockout mice) and demonstrate that the chemokine MCP-1 can up-regulate the innate immunity of AM. Innate immunity of the respiratory system in normal individuals is sufficient to prevent *P. aeruginosa* infection, but is unable to eradicate the bacteria in immunodeficient individuals, resulting in recurrent infection or acute dissemination. Activating the immunological functions of AECII may lend much-needed help in fighting microorganisms such as *P. aeruginosa*. The results from our studies demonstrate that MCP-1 production was dramatically increased after infection of AECII. Further, we have shown that direct application of synthetic MCP-1 peptide also boosts AM functions, albeit in a less potent way compared to induction by *P. aeruginosa*-infected medium. In addition, the instillation of MCP-1-neutralizing antibodies into mouse lungs blocks cytokine secretion and induces more severe pneumonia, indicating a critical functional role of this particular chemokine. The foremost function of these secreted factors is to recruit AM and boost their microbicidal activity. Importantly, we show direct evidence from MCP-1 knockout mice, which exhibited reduced clearance of *P. aeruginosa* and increased pathological alterations in the lungs. In contrast, reconstitution of MCP-1 using conditioned medium partially recovered the inadvertent pathophysiology induced by *P. aeruginosa*.

Lipid microdomains of cell surface make the platforms and initiate dynamics that directly or indirectly regulate the down-stream activities, including cytokine secretion by AECII and bacterial phagocytosis by AM. We showed that lipid rafts in AECII are reorganized following acute infection by *P. aeruginosa* [12], which is consistent with several studies regarding *P. aeruginosa* or other microorganisms [13]–[15]. Thus we reasoned that AECII initial contact with the invading *P. aeruginosa* occurs through lipid rafts. Raft signaling may be beneficial to the host against *P. aeruginosa* [18], although some reports indicate that other pathogens (e.g., HIV) may use lipid rafts to subvert the innate immunity functions [19]. We also suggest that lipid rafts may directly associate the signaling pathway in activating AM [17]. Using ImageJ software, we provide quantitative evidence that lipid raft aggregates have a specific role in host defense against infection. Our data indicate that ceramide only associates with *P. aeruginosa* adhesion, while cholesterol impacts both adhesion and internalization to AECII cells. Our study also substantiates the role of lipid rafts in cytokine secretion, consistent with previous work in this field showing that TNF-α secretion is mediated by recruiting syntaxin-4 to lipid rafts [20]. Previous studies showed that various mechanisms such as JNK, TNF-α and IL1-β are implicated in boosting MCP-1 secretion [21].

Importantly, we have identified that Lyn is crucial in host defense against bacterial infections. Lyn up-regulates MCP-1 secretion and promotes AM activity. Although Lyn was previously shown to be involved in the progression of Friend virus-induced erythroleukemia [22], Lyn's role in secretion of cytokines is not known. We also showed that the Lyn mediated regulation of MCP-1 secretion may be through the activation of NF-κB, which is a key transcriptional factor for inducing gene expression of many immune molecules including MCP-1. Our data are consistent with a report from Kowalski et al [13], who showed that *P. aeruginosa* infection of respiratory epithelial cells induced NF-κB nuclear translocation and blocking rafts using cholesterol chelating agents prevented this signaling. Lyn may also be able to increase reactive oxygen species production and release through the activation of PI3K and Akt [23]–[25] or other cellular signals (i.e., p47Phox, M.W., S.K., unpublished observations). Mechanistically, we identified a potential Lyn regulation site via H4 hyperacetylation at location K16 (unpublished). Additionally, we found that these responses may involve bacterial components including pili and ExoS. This is interesting because ExoS may be an important component of exoenzymes, responsible for intoxicating host defense mechanisms for bacterial benefit. In fact, pili as the surface structure of the bacterium may directly target lipid rafts to impact host signaling (not published). Also, reduction of MCP-1 production was seen when cells were challenged with a pili-deficient strain but increased MCP-1 secretion was noticed using the pili-corrected strain. Finally, our study also demonstrates that although dead bacteria or their partial products (LPS and supernatant) may have effects on igniting host defense, it is the live bacterium that stirred the strongest inflammatory response.

Whether or not AM are crucial for mitigating *P. aeruginosa* infection has been controversial. Our study has reaffirmed that AM are an important entity for exerting immune defense during acute *P. aeruginosa* infection. Our data indicates that activated AM are essential for phagocytosis of invading bacteria and scavenging dead bodies of neutrophils, evidenced by greater neutrophil penetration and pro-inflammatory cytokine response in Lyn^−/−^ mice. The increased superoxide in AECII-activated AM can be utilized to kill the engulfed bacteria, particularly important during high doses of bacterial infection. Our results indicate that AM alone may be less effective, while cytokines or chemokines derived from AECII or other unidentified mechanisms may give AM an added power that is required for clearance of invading microorganisms (Figure 7). However, the effects of AECII immune roles may impact other cell types such as neutrophils and lymphocytes, which may be interesting directions for future studies.

The Lyn axis maintains a fine balance involving several Src members or signaling proteins. Lyn through the ITAM motif up-regulates Syk function, which has been shown as important for opsonic phagocytosis by monocytes through FcγII mechanisms [26]. Lyn also has a positive role in ITIM motif that increases SH2 inositol phosphatase (SHIP), which may down-regulate inflammatory cytokines during acute infections [27]–[29]. Phagocytosis of the bacterium is dependent on many virulence factors as well as the host mediators. One of the major virulent effects of T3SS is disruption of cortical actin in phagocytic cells. We have reported earlier that a similar mechanism also exists in alveolar epithelium. Our recent study showed that T3SS is also involved in the phagocytosis of *P. aeruginosa* into AM [17]. *P. aeruginosa* releases these toxins directly into the host cytoplasm. Once activated, the ExoS and ExoT affect small GTPases by ADP ribosylation and GTPase activity, thereby deregulating cytoskeletal polymerization [30]. *P. aeruginosa* uses this mechanism to escape from phagocytosis and survive in the airways. Whether ExoS and ExoT have any direct effect on host cytokine secretion is less clear. Current studies demonstrate that secretion of MCP-1 may be inhibited by ExoS, as an ExoS-deficient strain induced even stronger secretion. Strain of ΔExoS also reduced superoxide release, but by an unknown mechanism.

Together, our study not only delineates the immunological role of AECII but also suggests that AM, aided by AECII, are important for combating *P. aeruginosa* infection. Specifically, we identified that the secreted MCP-1 was a major booster for AM defense function and was regulated by Lyn. Furthermore, our data suggest that both Lyn and MCP-1 have crucial roles in balancing inflammatory response to bacterial challenge through interaction with various cell signaling proteins. The results from the current studies may open new avenues for the development of much-needed therapeutics for treating *P. aeruginosa* infection.

## Methods

### 

#### Animals

C57BL6 mice were purchased from the Jackson Laboratory. Lyn^−/−^ mice based on J129/C57BL6 mice (6–8 weeks, female and male) were as described previously [31], [32]. MCP-1 mice were previously described [33], which were purchased from the Jackson Laboratory. The animal experiments have been approved by UND IACUC committee and were performed in accordance to the animal use and care guidelines. We anesthetized mice with 45 mg/kg ketamine plus 5 mg/kg diazepam, and intranasally instilled 0.1×10^7^ (PAK) to 0.5×10^8^ (PAO1) colony-forming units (CFUs) of *P. aeruginosa* for various times. After bronchoalveolar lavage (BAL), the trachea and lung were excised for homogenization or inflated with 50% OCT or formalin fixation. Intranasal instillation of the conditioned medium derived from *P. aeruginosa* infection with different bacterial strains was performed similarly as above. In selected experiments to check the efficiency of instillation, we used intratracheal instillation and ventilation procedures to confirm evenness of distribution in the lung.

#### Cell culture

Rat AECII cells were isolated as previously described and used within 2 days to preserve type II characteristics [12], [34], [35]. Mouse AM were isolated by bronchoalveolar lavage (BAL) concurrent to AECII isolation as described [17], [36], [37]. AECII cells were grown in Dulbecco's modified Eagle's medium (DMEM) and AM in RPMI 1640 medium supplemented with 10% newborn calf serum and penicillin/streptomicin antibiotics in 5% CO_2_ incubator. A549, MLE-12 and MHS cells were obtained from ATCC and maintained following the manufacture's instructions. For optimal seeding density, the cells were counted using neubauer chamber and dead cells were excluded by the trypan blue exclusion assay. We generated and collected conditioned medium from primary mouse AECII following PAO1 infection for 1 h and cultured another 24 h (after adding 100 µg/ml polymycin to kill the surface bacteria). The control medium without infection was collected for use. After co-culturing primary AM with the conditioned medium for 24 h, AM activity was detected by functional measurements as described in the related sections.

#### Bacterial strains and infection

PAO1 wt is with an intact *pilC* gene and thus with twitching motility provided by Dr. S. Lory (Harvard) [38]. PAO1 wt GFP, *ΔfliC* (flagellin minus), Δ*exo*S, Δ*exo*T and Δ*exo*U deletion mutant strains, PAK (pili wt), PAK *ΔpilC* (pili deficient) and PAO1 were obtained from Dr. Gerald Pier (Harvard Medical School) [39]. PA14 wt (Δ*exo*S, Δ*exo*T, Δ*exo*U and Δ*exo*Y) were from Dr. E. Drenkard (Massachusetts General Hospital) [40] and used in selected experiments. Bacteria were grown overnight in LB medium at 37°C without shaking. Absorbance at 600 nm was measured for quantification (0.1 OD = 1×10^8^ bacterium/ml).

#### Immunohistochemistry and immunocytochemistry staining and confocal imaging

Adherent cells in Labtek chamber slides or animal/human tissue sections were stained for cell surface markers using appropriate primary antibodies and double stained for immunofluorescence with TRITC or FITC conjugated respective secondary antibodies. The cells were washed, and pictures taken with Zeiss LSM 510 confocal laser scanning microscope (Carl Zeiss MicroImaging, Inc, Thornwood, NY). The human samples were used in accordance to the NIH exempt guidelines and approved by the UND IRB committee. For live cell imaging, MatTek glass bottom dishes were used with a portable incubation chamber to keep cells at 37°C.

### RT-PCR analysis

RNA was extracted from Lung homogenates and cells with Trizol (Invitrogen Corporation) according to the manufacturer's instructions. For detected genes, reverse transcription (RT) was performed using 1.5 mg of RNA, RNase ribonuclease inhibitor, Oligo dT and cloned AMV reverse transcriptase (Invitrogen Corporation). cDNA was amplified using gene-specific primers as summarized in **Table 2**. PCR products were separated by 1.0% agarose gel electrophoresis containing ethidium bromide and visualized under UV light. The results for each gene were normalized in comparison with GAPDH expression.

**Table 2:** Primers for cytokines.

Gene	Sense	Antisense
IL-6	TTGCCTTCTTGGGACTGATGCT	GTATCTCTCTGAAGGACTCTGG
IFN-γ	TGCATCTTGGCTTTGCAGCTCTTC	GGGTTGTTGACCTCAAACTTGGCA
TNF-α	ACCGTCAGCCGATTTGCTATCTCA	TGGACATTCGAGGCTCCAGTGAAT
IRF-1	CACCATGCCAATCACTCG	GGG TAG AGCTGCTGAGTCCA
MCP-1	TCCCAATGAGTAGGCTGGAGAGC	CAGAAGGCTTGAGGTGGTTGTG
GAPDH	TAAAGGGCATCCTGGGCTACACT	TTACTCCTTGGAGGCCATGTAGG

### Cytokine Immunoassay

Cytokine concentrations were measured by ELISA kit (eBioscience company) in cell culture medium, BAL fluid and lung homogenates collected at indicated times after infection. Briefly, the cells were treated as described as above. On 24 h or 48 h, culture mediums were collected and cells. For BAL fluid, the trachea was surgically exposed and cannulated, lungs were lavaged 5 times with 1.0 ml volumes of lavage fluid, the lavageates were pooled, and cells were removed by centrifugation. For lung homogenates, excised lungs were ground using a sterile glass-Teflon® in 500 µl PBS. 96-well plates (Corning Costar 9018) were coated with 100 µl/well of capture antibody in coating buffer and incubated overnight at 4°C. 100 µl aliquots of serum samples were added to the coated microtiter wells. The cytokine concentrations were determined with corresponding detection HRP-conjugated antibodies. The values were read at 450 nm and analyzed.

### Western blot and Co-immunoprecipitation

The sample from cells and lung homogenates were lysed and quantitated. The lysates were boiled for 5 min, and protease inhibitors added. The supernatants were collected, and 30 µg of each sample were loaded onto 10% SDS-polyacrylamide mini-gels and electrophoresed to resolve proteins. The proteins were then transferred to polyvinylidene difluoride membranes (Pierce Biotechnology) and blocked overnight at 4°C using 5% non-fat milk blocking buffer western antibody buffer [41], [42]. Membranes were incubated overnight at 4°C with the appropriate first antibodies diluted 1∶1,000 in 5% non-fat milk blocking buffer. After washing three times with washing solution, the antigen-antibody complexes were incubated for 2 h at room temperature with horseradish peroxidase-conjugated secondary antibody (Amersham) diluted 1∶2,000. Signals were visualized using enhanced chemiluminescence detection kit (SuperSignal West Pico; Pierce).

For co-immuno-precipitation, the supernatants were pre-cleared with bare protein A/G-Sepharose beads (Pierce) for 1 h at 4°C and then incubated with anti-MCP-1, anti-NFκB, or anti-Lyn Abs (Santa Cruz) bound to protein A/G Sepharose beads overnight at 4°C. The next day, beads were washed three times in lysis buffer and boiled in SDS sample loading buffer. The proteins were separated and analyzed by western blotting.

#### AM migration assay

AM migration was evaluated in either 8 µm transwell cell culture insert or chemotaxis chamber as described previously [43] using Boyden chamber. Briefly, cells were plated on the upper chamber and allowed to adhere overnight. After treatment, the filter was washed in phosphate buffered saline (PBS) and turned upside down; the migrated cells in the bottom of the filter were counted using specific red fluorescence (DiI, Molecular probes) staining followed by confocal microscopy. Cells were visualized by exciting with 533 nm wavelength HeNe laser and emission detected in the red spectra with a long pass filter. At least 100 fields per sample were counted for statistical purpose.

#### Fluorescent phagocytosis assay

AM cells were plated in 96 well plates and grown overnight. The cells were treated with the conditioned medium from AEC II for 2 h. Then FITC labeled *E. coli* or GFP PAO1 was used to infect the cells at MOI 1∶10. After 1 h incubation at 37°C the wells were washed and treated with Polymixin 100 µg/ml for 1 hr to kill any remaining extracellular bacteria. The number of phagocytosed bacteria was counted using Synergy HT fluorimeter (BioTek) with 485±20 nm excitation and 528±20 emission filters. Background correction was done for autofluorescence.

#### Scanning electron microscopy (SEM)

SEM was used to study morphology. AECII and AM cells were plated on collagen coated coverslips in 24-well plates. The cells were fixed in Karnovsky's fixative overnight, dehydrated by sequential alcohols, sputter coated with gold – palladium and images taken at 10000× magnification.

#### Lung edema

After infection, lungs were removed from the thoracic cavity, and the inferior third of the left lung were weighed and then placed in a drying oven at 90°C for 24 h. The specimen was reweighed, and the ratio of the weight before and after drying calculated.

#### Lipid peroxidation assay

Malondialdehyde (MDA) is an end product of lipid peroxidation process, and was measured in a colorimetric assay (Calbiochem, San Diego, CA) according to the manufacturer's instructions. Homogenized lung tissue in 62.5 mM Tris-HCL (pH = 6.8) supplemented with Complete-Mini Protease Inhibitor (Roche Diagnostics) in equal protein amounts were used in the assay.

#### Lung myeloperoxidase (MPO) assay

MPO assay was performed as described [44]. Samples were homogenized in 50 mM hexadecyltrimethylammonium bromide (HTAB) 50 mM KH_2_PO_4_, pH 6.0, and 0.5 mM EDTA, 1 mL/100 mg tissue and centrifuged for 15 min at 12000 rpm at 4°C. Supernatants were decanted and 100 µl of reaction buffer (0.167 mg/mL *O*-dianisidine, 50 mM KH_2_PO_4_, pH 6.0 and 0.0005% mM H_2_O_2_) was added to 100 µl sample. Absorbance was read at 460 nm at 2 min intervals.

#### H2DCF Assay for Superoxide production

H2DCF dye (Molecular Probes) does not normally fluoresce and emits green fluorescence upon reaction with superoxide inside cells[45]. Cells were treated as above and equal amount(s) of dye added. Fluorescence was measured after 10 min incubation using the fluorimeter (BioTek, in our laboratory). For confirmation, NBT assay was used as we described previously [17].

#### siRNA

A pool of four specific siRNAs or sense controls was (‘A pool’ is singular) purchased from Dharmacon (West Lafayette, Colorado) to deplete Lyn in MLE-12, AECII and MSH cells and the depleted expression assessed as we previously described [17].

#### Statistical analyses

Comparison of test with controls was done in triplicates and the results were analyzed using two tailed Student's *t* test with a P value<0.05. For multiple groups with an equal variance, we performed one-way ANOVA. For analyzing image data the sample size was increased by taking pictures of at least 100 cells per sample.

## Supporting Information

Figure S1 AECII cells activate macrophages after PAO1 infection. Actin polymerization was induced by the medium from A549 cells infected by PAO1 in the Boyden chamber, which has two compartments separated by a porous membrane. RAW264 cells were seeded in the top and A549 cells in the bottom. Infection of A549 cells was done separately before inoculation into the chamber. Migrating cells were identified by staining the porous membrane with rhodamine phalloidin for actin and DAPI for nucleus and images taken by Zeiss confocal microscope. RAW264 cells started migrating towards infected A549 cells within 30 min (A). Quantification of actin cytoskeletal changes in RAW264 cells upon infection (B). RAW264 cells were grown on coverslips in 24 well plate. Cells were infected with PAO1 at 1∶10 ratio for 1 h. The cells were fixed in 4% PFA and stained with FITC CT (Lipid raft marker) and Rhodamine Phalloidin (Actin). Untreated controls showed more number of cells (52%) with cytoskeletal changes like lamellipodium formation. The graph shows percent positive for cytoskeletal changes and error bar denotes standard deviation (P<0.01). AM conditioned medium has less potential for attracting AM than the conditioned medium from AECII by determining the migration index (C). Percentage of the migration of treated samples against total cells counted.
(1.33 MB TIF)

Figure S2 MCP-1 secreted by MLE-12 or isolated AECII is a major chemokine by PAO1 infection. MCP-1 expression is induced in MLE-12 cells by PAO1 infection. Lyn siRNA and various inhibitors (PP2 and Rac1 inhibitor, Calbiochem) decrease the expression of MCP-1 (A). AM under infection show less secretion of MCP-1 than AECII cells. Also, additional inhibitors examined demonstrate inhibition of MCP-1 expression in AM and AECII cells. In addition, co-culturing of AM with AECII induces increased secretion of MCP-1 than either cell alone (B).
(1.16 MB TIF)

Figure S3 Activated AECII demonstrate increased immunological characteristics including class II expression under PAO1 infection (A). Immunological markers including IL-12R (FITC) and IL-17R (TRITC) are increased against controls (not shown) under PAO1 infection (B) (all antibodies obtained from Santa Cruz).
(1.59 MB TIF)
