# Supplementary figures and images for: Alveolar Epithelial Type II Cells Activate Alveolar Macrophages and Mitigate P. Aeruginosa Infection
Source: PLoS One. 2009 Mar 23;4(3):e4891. doi: 10.1371/journal.pone.0004891 (PMC2654511; doi:10.1371/journal.pone.0004891)

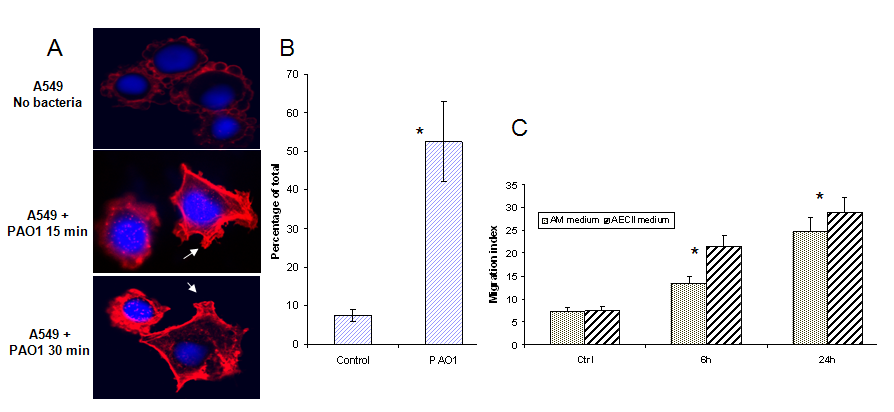

Supplement: Figure S1 — AECII cells activate macrophages after PAO1 infection. Actin polymerization was induced by the medium from A549 cells infected by PAO1 in the Boyden chamber, which has two compartments separated by a porous membrane. RAW264 cells were seeded in the top and A549 cells in the bottom. Infection of A549 cells was done separately before inoculation into the chamber. Migrating cells were identified by staining the porous membrane with rhodamine phalloidin for actin and DAPI for nucleus and images taken by Zeiss confocal microscope. RAW264 cells started migrating towards infected A549 cells within 30 min (A). Quantification of actin cytoskeletal changes in RAW264 cells upon infection (B). RAW264 cells were grown on coverslips in 24 well plate. Cells were infected with PAO1 at 1∶10 ratio for 1 h. The cells were fixed in 4% PFA and stained with FITC CT (Lipid raft marker) and Rhodamine Phalloidin (Actin). Untreated controls showed more number of cells (52%) with cytoskeletal changes like lamellipodium formation. The graph shows percent positive for cytoskeletal changes and error bar denotes standard deviation (P<0.01). AM conditioned medium has less potential for attracting AM than the conditioned medium from AECII by determining the migration index (C). Percentage of the migration of treated samples against total cells counted. (1.33 MB TIF) [file pone.0004891.s001.tif]

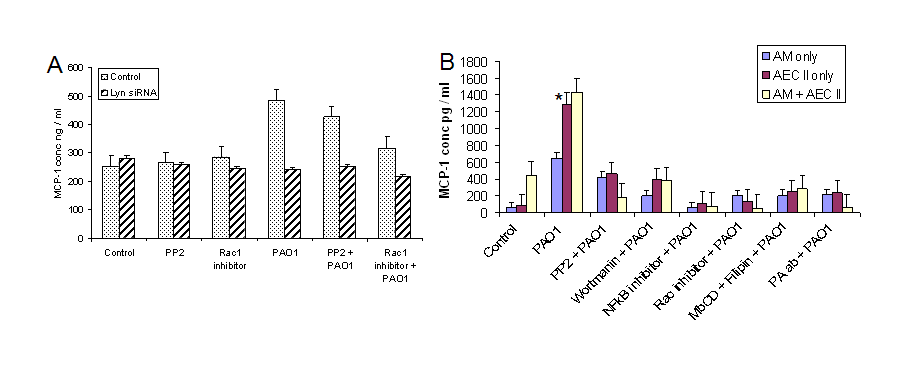

Supplement: Figure S2 — MCP-1 secreted by MLE-12 or isolated AECII is a major chemokine by PAO1 infection. MCP-1 expression is induced in MLE-12 cells by PAO1 infection. Lyn siRNA and various inhibitors (PP2 and Rac1 inhibitor, Calbiochem) decrease the expression of MCP-1 (A). AM under infection show less secretion of MCP-1 than AECII cells. Also, additional inhibitors examined demonstrate inhibition of MCP-1 expression in AM and AECII cells. In addition, co-culturing of AM with AECII induces increased secretion of MCP-1 than either cell alone (B). (1.16 MB TIF) [file pone.0004891.s002.tif]

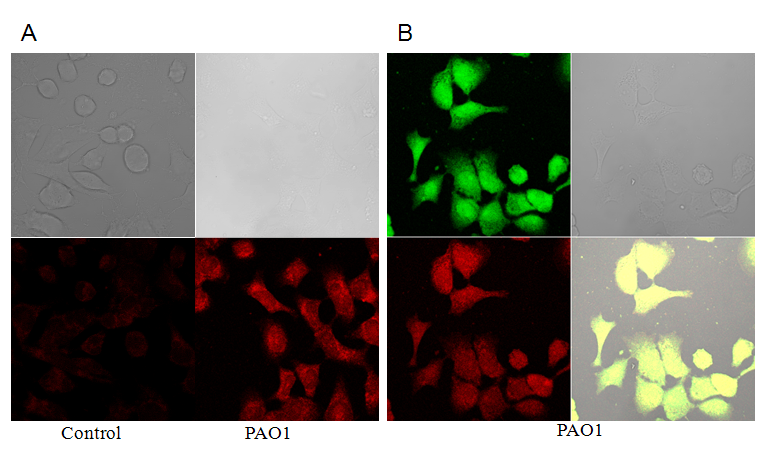

Supplement: Figure S3 — Activated AECII demonstrate increased immunological characteristics including class II expression under PAO1 infection (A). Immunological markers including IL-12R (FITC) and IL-17R (TRITC) are increased against controls (not shown) under PAO1 infection (B) (all antibodies obtained from Santa Cruz). (1.59 MB TIF) [file pone.0004891.s003.tif]
